# Overweight during lactation and its implications for biometric, nutritional and cardiovascular parameters of young and adult male and female rats

**DOI:** 10.1017/jns.2020.21

**Published:** 2020-07-06

**Authors:** Gracielle Amaral de Araújo, Raysa da Silva Farias, Samuel de Sousa Pedro, Nazareth N. Rocha, Fernanda C. F. Brito, Christianne B. V. Scaramello

**Affiliations:** Laboratory of Experimental Pharmacology, Fluminense Federal University, Niterói, Rio de Janeiro, Brazil

**Keywords:** Lactation, Child development, Overweight, Cardiovascular system, Sex characteristics, AC, abdominal circumference, IVS, interventricular septum thickness, IVSd, interventricular septum thickness diastole, IVSs, interventricular septum thickness systole, LVID, left ventricle internal diameter, LVIDd, left ventricle internal diameter diastole, LVPW, left ventricle posterior wall thickness, LVPWd, left ventricle posterior wall thickness diastole, LVPWs, left ventricle posterior wall thickness systole, NAL, nose-to-anus length, TC, thoracic circumference

## Abstract

Litter size reduction can induce early overnourishment, being an attractive experimental model to study short- and long-term consequences of childhood obesity. Epidemiological data indicate sex differences regarding cardiometabolic disorders and hypertrophic cardiomyopathy. The present study aimed to describe biometric, nutritional and cardiovascular changes related to neonatal overweight promoted by litter size reduction in young and adult Wistar rats of both sexes. Litter adjustment to eight or four pups/mother (1:1 male-to-female ratio) gave, respectively, control and overweight groups. Body mass, food intake, haemodynamic and echocardiographic parameters and cardiorespiratory capacity were evaluated at postnatal days 30 and 150. Diminished litters were correlated with higher body mass and weight gain (12 %) during lactation, validating the experimental model of neonatal overweight. Soon after weaning male (16 %) and female (25 %) offspring of these litters presented a lower food intake than their respective control, without differences in body mass. Adult males from reduced litters presented higher abdominal circumference (7 %), systolic blood pressure (10 %), interventricular septum thickness (15 %) and relative wall thickness (15 %) compared with their respective control. Rats' performance on the maximal effort ergometer test was not affected by neonatal overweight. Data suggest the occurrence of catch-down growth and hypophagia in male and female rats submitted to neonatal overweight. However, only male rats presented haemodynamic and cardiac structural changes. These findings are crucial to personalised/gender medicine.

Obesity/overweight is a major global health problem that leads to increased mortality. This condition in early life may be related to postnatal nutrition and can evoke metabolic disorders and several co-morbidities, increasing cardiovascular risk and favouring CVD in adulthood. Estimates of deaths related to CVD increased about 14 % between 2006 and 2016^(1–4)^.

Studies investigating the relationship between events in early life, as nutritional insults, and functional status in the future belong to a new research field named ‘developmental origin of health and disease’ (DOHaD). The history of DOHaD as a research field reached a milestone with David Barker's theory encompassing the programming of diseases with fetal origins^(5)^. The understanding that the environment and individual lifestyle directly interact with the genome to influence epigenetic changes is growing fast^(6)^. These changes alter homeostasis through the remodelling of organs and tissues^(7)^. As the heart is not entirely developed soon after birth, nutritional insults in early life may contribute to the occurrence of cardiac diseases in adulthood also through direct effects^(8)^.

Animal models comprise an interesting strategy to evaluate future outcomes related to nutritional insults in early life and developmental plasticity. Studies with male animals (mice and rats) report that overnourishment during lactation induces metabolic and haemodynamic heart impairment during adulthood. In general, experimental models of neonatal overfeeding encompass litter size reduction that allows milk supply increase to the offspring. This experimental model is cheap and effective to investigate short- and long-term consequences of neonatal overweight^(9–12)^. However, such evidence is not available for female animals.

Despite the accumulating evidence that sex leads to differences in biology, for several reasons, the variable sex has been largely ignored in biomedical research^(13)^. In humans, there are sex differences regarding CVD. The literature points out sex differences in cardiometabolic disorders and differences between men and women with hypertrophic cardiomyopathy^(14,15)^. Individualised medicine must consider sex and gender to initiate personalising care, allowing the improvement of the outcomes. For this, evidence supporting sex-specific decisions also needs to be provided by basic scientists^(16)^. Thus, the present study aimed to evaluate biometric, nutritional and cardiovascular outcomes related to neonatal overweight/overnourishment in young and adult Wistar rats of both sexes.

## Materials and methods

### Animals and experimental model

The Ethics Committee of Fluminense Federal University (Niteroi, Brazil) approved the use of animals (Comissão De Ética No Uso De Animais (CEUA) UFF812/2016) following the Guide for the Care and Use of Laboratory Animals (National Institutes of Health (NIH) publication no. 8023, revised 1978). All rats received standard chow (Nuvilab^®^) and water *ad libitum* at controlled conditions (22°C, 55–65 % humidity, 12 h light–12 h dark cycle). The breeding laboratory of the University provided Wistar rats used for mating (F0 generation). Male (*n* 10) and female rats (*n* 20) about 3 months of age and no kinship were mated (two females for one male) for 5 d. Pregnant rats placed in individual cages gave birth to ten to twelve pups after 21 d of gestation. The offspring (F1 generation) were divided into two groups at postnatal day 1 to minimise stress by simple randomisation^(17)^:

Control – eight pups per mother (four males and four females);

Overweight – four pups per mother (two males and four females).

There was a total of seventy-two rats from the F1 generation:

Control – thirty-two animals (sixteen males and sixteen females) – four litters;

Overweight – forty animals (twenty males and twenty females) – ten litters.

Offspring analysis occurred at postnatal days 30 and 150, being considered young and adult animals^(18)^. Whenever possible, data were collected precisely from the same rats at both ages. Euthanasia happened at the end of the experimental period after administrating a lethal dose of thiopental intraperitoneally.

### Biometric and nutritional analyses

Body mass was monitored from birth to postnatal day 150, while food intake monitoring began upon weaning at postnatal day 21, allowing biometric and nutritional analysis^(19,20)^.

Feed efficiency was estimated between postnatal days 21–30, 30–150 and 21–150, using the formula: (final body mass – initial body mass)/Σfood intake.

It was possible to record other biometric parameters of anaesthetised rats using a tape measure: nose-to-anus length (NAL), abdominal circumference (AC) and thoracic circumference (TC) (cm).

BMI was calculated through the formula: body mass/NAL^2^.

It was possible to achieve complete biometric and nutritional data from eight animals/group at both ages.

### Echocardiography studies

The analyses of cardiac structure and function were performed through transthoracic echocardiography using a portable ultrasound system equipped with a 10 MHz transducer (Siemens Accusion Cypress). Previously the animals were anaesthetised with ketamine plus xylazine (50 mg + 5 mg/kg intraperitoneally). The assays were performed according to the American Society of Echocardiography^(21)^ and all parameters were measured at least three times per animal. The parameters recorded to address cardiac structure were left ventricular internal diameter (LVID), interventricular septum thickness (IVS) and left ventricular posterior wall thickness (LVPW), measured in systole and diastole, as well as relative wall thickness, left ventricle mass, and left atrium:aorta ratio. Systolic volume, ejection fraction and fractional shortening, related to functional parameters, were calculated through algorithms of the equipment software. The parameter recorded to evaluate diastolic function was mitral deceleration time. It was possible to achieve complete echocardiographic data from at least ten animals per group at both ages.

### Haemodynamic evaluation

Haemodynamic evaluation was performed by indirect measurement of systolic blood pressure and heart rate through the tail-cuff method^(22,23)^. The assays occurred in the morning after 3 d of acclimatisation using the ADInstruments ML125 NIBP (Non-Invasive Blood Pressure) system connected to the ADInstruments PowerLab/400 digital–analogue converter. The signal was analysed using LabChart 6 Pro software (ADInstruments). Final systolic blood pressure and heart rate values of each animal were calculated by taking the average of six successful separate measurements obtained in the absence of spontaneous tail movement in awake rats.

Thus, because of the assay's stress bias, it was not possible to record haemodynamic parameters of all animals submitted to echocardiography. It was possible to achieve complete haemodynamic data from eight animals/group, preferably at both ages.

### Maximal effort ergometer test

After 3 d of acclimatisation, responsive animals (non-sedentary) were also submitted to a maximum effort ergometer test (day 4). Non-responsive animals (sedentary) were discarded from this test. Thus, it was not possible to evaluate all rats submitted to previous assays. Data were achievable from at least five animals per group, preferably at both ages.

The protocol comprised a treadmill (Imbrasport^®^), without inclination and initial speed of 0⋅9 km/h, followed by progressive increments of 0⋅3 km/h every 3 min until animals were considered to be exhausted. The end of the test was determined when the animals remained still for at least 10 s. The parameters recorded were distance travelled, time spent and maximum speed developed in the test^(24,25)^.

### Statistical analysis

The Kolmogorov−Smirnov test was applied to verify normality and data were expressed as mean vales and standard deviations. Body mass recorded throughout lactation was analysed using a two-way ANOVA. The tested factors were litter size *v.* time. As the interaction was significant, the simple effects were analysed by Bonferroni's *post hoc* test for multiple comparisons between control and overweight groups within the same sex. The unpaired *t* test was used to compare data obtained from these groups after weaning at the same age as well as weight gain during lactation. Statistical analyses were performed using Prism Software (Graph Pad Prism 7.0). A value of *P* < 0⋅05 was considered statistically significant.

## Results

### Body and nutritional analysis

**Fig. 1.**
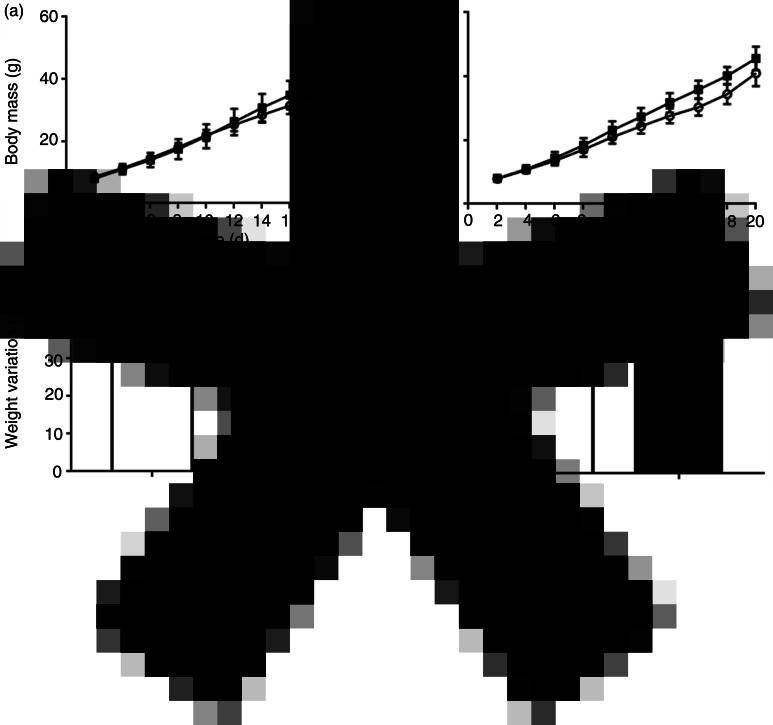
Body mass (a, b) and weight gain (c, d) in grams throughout lactation. (a, c) Male offspring. (b, d) Female offspring. -○-, □, Control group (*n* 16); -■-, ■, overweight group (*n* 20). Values are means, with standard deviations represented by vertical bars. Body mass data were analysed using two-way ANOVA followed by Bonferroni's *post hoc* test. Weight gain was analysed using the unpaired *t* test. * *P* < 0⋅05 *v*. respective control group.

Figs 1(a) and 1(b) show the body mass of male and female offspring throughout lactation. Reduced litters presented higher body mass during lactation and increased weight gain (Fig. 1(c) and 1(d)). Similar values of body mass, NAL, TC and BMI were seen between groups within the same sex at postnatal days 30 and 150. Nevertheless, adult males from reduced litters presented higher AC and AC:TC ratio than those from normal ones (Tables 1 and 2).

**Table 1.** Biometric parameters of male offspring (Mean values and standard deviations)

	Postnatal day 30				Postnatal day 150			
	Control		Overweight		Control		Overweight	
	Mean	sd	Mean	sd	Mean	sd	Mean	sd
*n*	8		8		8		8	
BM (g)	165⋅0	10⋅4	158⋅9	16⋅5	448⋅4	21⋅1	458⋅9	66⋅4
NAL (cm)	17⋅3	0⋅8	16⋅9	0⋅6	25⋅8	1⋅1	25⋅5	1⋅1
BMI (g/cm^2^)	0⋅55	0⋅05	0⋅56	0⋅03	0⋅67	0⋅03	0⋅70	0⋅07
AC (cm)	15⋅8	1⋅1	15⋅3	0⋅9	20⋅5	1⋅4	22⋅1*	1⋅3
TC (cm)	13⋅8	0⋅8	13⋅7	0⋅5	17⋅8	1⋅3	18⋅2	0⋅6
AC:TC	1⋅15	0⋅05	1⋅12	0⋅02	1⋅15	0⋅04	1⋅21*	0⋅06

BM, body mass; NAL, nose-to-anus length; AC, abdominal circumference; TC, thoracic circumference.

* *P* < 0⋅05 *v*. respective control group. Data were analysed using the unpaired *t* test.

**Table 2.** Biometric parameters of female offspring (Mean values and standard deviations)

	Postnatal day 30				Postnatal day 150			
	Control		Overweight		Control		Overweight	
	Mean	sd	Mean	sd	Mean	sd	Mean	sd
*n*	8		8		8		8	
BM (g)	129⋅4	14⋅8	138⋅2	13⋅0	248⋅8	19⋅2	260⋅7	31⋅7
NAL (cm)	16⋅5	0⋅6	16⋅6	0⋅4	21⋅7	0⋅9	22⋅3	0⋅7
BMI (g/cm^2^)	0⋅48	0⋅04	0⋅50	0⋅04	0⋅53	0⋅03	0⋅53	0⋅06
AC (cm)	14⋅0	0⋅8	14⋅8	0⋅8	18⋅0	0⋅6	18⋅6	0⋅9
TC (cm)	12⋅8	0⋅9	13⋅1	0⋅6	15⋅3	0⋅8	15⋅4	0⋅8
AC:TC	1⋅09	0⋅04	1⋅13	0⋅05	1⋅18	0⋅04	1⋅21	0⋅05

BM, body mass; NAL, nose-to-anus length; AC, abdominal circumference; TC, thoracic circumference.

Despite no differences in feed efficiency, food intake was found lower in rats from reduced litters compared with those from regular litters soon after weaning. In the same period, females from reduced litters presented lower weight gain than their respective controls (Tables 3 and 4).

**Table 3.** Nutritional parameters of male offspring (Mean values and standard deviations)

	Postanatal days 21–30				Postanatal days 30–150				Postnatal days 21–150			
	Control		Overweight		Control		Overweight		Control		Overweight	
	Mean	sd	Mean	sd	Mean	sd	Mean	sd	Mean	sd	Mean	sd
*n*	8		8		8		8		8		8	
Weight variation (g)	46⋅9	14⋅8	40⋅7	9⋅1	335⋅9	20⋅7	345⋅0	64⋅6	382⋅8	24⋅0	385⋅7	57⋅3
Food intake (g)	131⋅3	16⋅4	110⋅3*	20⋅4	2540	232⋅5	2663	168⋅2	2672	216⋅5	2773	148⋅7
Feed efficiency	0⋅350	0⋅066	0⋅369	0⋅043	0⋅133	0⋅014	0⋅129	0⋅019	0⋅145	0⋅018	0⋅139	0⋅015

* *P* < 0⋅05 *v*. respective control group. Data were analysed using the unpaired *t* test.

**Table 4.** Nutritional parameters of female offspring (Mean values and standard deviations)

	Postnatal days 21–30				Postnatal days 30–150				Postnatal days 21–150			
	Control		Overweight		Control		Overweight		Control		Overweight	
	Mean	sd	Mean	sd	Mean	sd	Mean	sd	Mean	sd	Mean	sd
*n*	8		8		8		8		8		8	
Weight variation (g)	42⋅0	6⋅5	33⋅9	7⋅1	161⋅7	7⋅6	172⋅0	14⋅4	203⋅8	12⋅8	205⋅9	9⋅9
Food intake (g)	137⋅9	8⋅5	102⋅8	23⋅1	1916	357⋅2	1895	99⋅8	2054	348⋅7	1998	103⋅2
Feed efficiency	0⋅304	0⋅037	0⋅331	0⋅020	0⋅087	0⋅017	0⋅091	0⋅006	0⋅102	0⋅020	0⋅103	0⋅004

### Haemodynamic and echocardiographic parameters

Tables 5 and 6 show haemodynamic and echocardiographic parameters from male and female animals, respectively. Male rats from reduced litters presented higher systolic blood pressure and structural changes in youth (as higher IVSd, IVSs, LVPWd, LVPWs and LMV) and adulthood (higher IVSd and relative wall thickness, lower LVIDd) than from regular ones (Table 5). Curiously, adult female rats from reduced litters presented lower systolic blood pressure compared with their respective controls. They also presented structural changes characterised by an increased IVSd, LVPWd and relative wall thickness in youth without functional alterations (Table 6). All animals presented ejection fraction superior to 80 % and similar values of mitral deceleration time.

**Table 5.** Haemodynamic and echocardiographic data of male offspring (Mean values and standard deviations)

	Postnatal day 30				Postnatal day 150			
	Control		Overweight		Control		Overweight	
	Mean	sd	Mean	sd	Mean	sd	Mean	sd
Haemodynamic data								
*n*	8		11		10		10	
Systolic blood pressure (mmHg)	100⋅6	5⋅2	111⋅6**	6⋅5	126⋅3	15⋅9	142⋅6*	15⋅2
Heart rate (bpm)	364⋅2	61⋅0	363⋅4	58⋅1	332⋅0	60⋅2	341⋅6	48⋅4
Echocardiographic data								
*n*	12		11		10		10	
IVSd (cm)	0⋅112	0⋅009	0⋅127*	0⋅004	0⋅195	0⋅025	0⋅227*	0⋅028
IVSs (cm)	0⋅190	0⋅031	0⋅227*	0⋅018	0⋅338	0⋅040	0⋅354	0⋅052
LVIDd (cm)	0⋅335	0⋅037	0⋅379	0⋅056	0⋅553	0⋅103	0⋅525	0⋅054
LVIDs (cm)	0⋅122	0⋅017	0⋅145	0⋅043	0⋅238	0⋅085	0⋅191	0⋅064
LVPWd (cm)	0⋅112	0⋅010	0⋅127*	0⋅004	0⋅199	0⋅016	0⋅230	0⋅029
LVPWs (cm)	0⋅191	0⋅029	0⋅237*	0⋅020	0⋅332	0⋅029	0⋅359	0⋅045
RWT (cm)	0⋅675	0⋅074	0⋅679	0⋅091	0⋅740	0⋅139	0⋅880*	0⋅115
LVM (g)	0⋅709	0⋅029	0⋅768*	0⋅058	1⋅170	0⋅166	1⋅271	0⋅200
LA:Ao	1⋅050	0⋅106	1⋅118	0⋅143	0⋅943	0⋅140	1⋅025	0⋅062
LVEF (%)	94⋅61	2⋅43	93⋅54	3⋅27	88⋅09	8⋅57	95⋅48*	3⋅85
FS (%)	64⋅04	6⋅02	63⋅08	6⋅19	58⋅29	9⋅28	69⋅58*	9⋅47
Mitral DT (ms)	62⋅75	6⋅84	67⋅67	8⋅99	89⋅63	12⋅18	88⋅13	6⋅64

IVSd, interventricular septum thickness diastole; IVSs, interventricular septum thickness systole; LVIDd, left ventricle internal diameter diastole; LVIDs, left ventricle internal diameter systole; LVPWd, left ventricle posterior wall thickness diastole; LVPWs, left ventricle posterior wall thickness systole; RWT, relative wall thickness; LVM, left ventricle mass; LA:Ao, left atrium:aorta ratio; LVEF, left ventricle ejection fraction; FS, fractional shortening; mitral DT, mitral deceleration time.

* *P* < 0⋅05 *v*. respective control group. Data were analysed using the unpaired *t* test.

**Table 6.** Haemodynamic and echocardiographic data of female offspring (Mean values and standard deviations)

	Postnatal day 30				Postnatal day 150			
	Control		Overweight		Control		Overweight	
	Mean	sd	Mean	sd	Mean	sd	Mean	sd
Haemodynamic data								
*n*	8		11		10		10	
Systolic blood pressure (mmHg)	101⋅5	7⋅3	106⋅6	3⋅9	140⋅5	19⋅6	124⋅8*	15⋅3
Heart rate (bpm)	406⋅2	36⋅5	362⋅8	62⋅6	350⋅6	41⋅3	353⋅9	49⋅4
Echocardiographic data								
*n*	12		11		10		10	
IVSd (cm)	0⋅116	0⋅006	0⋅128*	0⋅005	0⋅158	0⋅019	0⋅177	0⋅032
IVSs (cm)	0⋅217	0⋅037	0⋅212	0⋅014	0⋅302	0⋅020	0⋅315	0⋅037
LVIDd (cm)	0⋅358	0⋅059	0⋅350	0⋅024	0⋅535	0⋅059	0⋅504	0⋅062
LVIDs (cm)	0⋅149	0⋅076	0⋅127	0⋅024	0⋅191	0⋅063	0⋅160	0⋅039
LVPWd (cm)	0⋅115	0⋅006	0⋅128*	0⋅005	0⋅165	0⋅022	0⋅179	0⋅030
LVPWs (cm)	0⋅212	0⋅029	0⋅214	0⋅013	0⋅300	0⋅024	0⋅315	0⋅032
RWT (cm)	0⋅654	0⋅088	0⋅733*	0⋅038	0⋅629	0⋅135	0⋅721	0⋅155
LVM (g)	0⋅725	0⋅039	0⋅750	0⋅041	1⋅000	0⋅074	1⋅050	0⋅144
LA:Ao	1⋅075	0⋅108	1⋅074	0⋅129	0⋅960	0⋅112	1⋅049	0⋅154
LVEF (%)	95⋅18	1⋅85	93⋅96	3⋅87	93⋅81	4⋅46	94⋅91	3⋅52
S (%)	65⋅88	4⋅13	64⋅04	6⋅19	65⋅12	7⋅19	66⋅12	9⋅23
Mitral DT (ms)	62⋅50	7⋅15	67⋅75	8⋅07	87⋅92	8⋅31	90⋅00	4⋅63

IVSd, interventricular septum thickness diastole; IVSs, interventricular septum thickness systole; LVIDd, left ventricle internal diameter diastole; LVIDs, left ventricle internal diameter systole; LVPWd, left ventricle posterior wall thickness diastole; LVPWs, left ventricle posterior wall thickness systole; RWT, relative wall thickness; LVM, left ventricle mass; LA:Ao, left atrium:aorta ratio; LVEF, left ventricle ejection fraction; FS, fractional shortening; mitral DT, mitral deceleration time.

* *P* < 0⋅05 *v*. respective control group. Data were analysed using the unpaired *t* test.

### Performance on maximal effort ergometer test

**Fig. 2.**
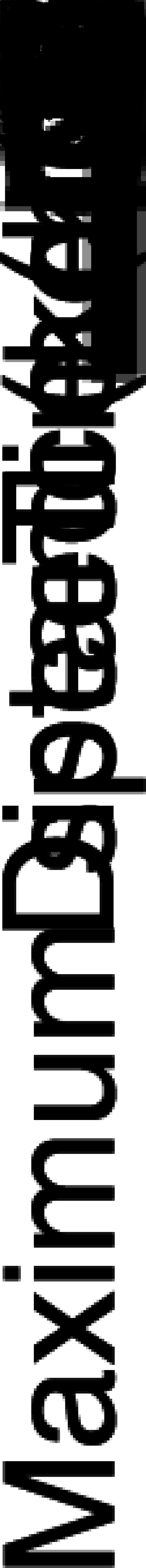
Data from the maximal effort ergometer test (a−f) at postnatal days (PND) 30 and 150. (a–c) Male offspring: control (□; *n* 5); overweight (■; *n* 8). (d–f) Female offspring: control (□; *n* 8); overweight (■; *n* 7). (a, d) Time spent (h). (b, e) Distance travelled (km). (c, f) Maximum speed developed (km/h). Values are means, with standard deviations represented by vertical bars. Data from the maximal effort ergometer tests were analysed using the unpaired *t* test. * *P* < 0⋅05 *v*. respective control group.

Overweight and control groups of male and female offspring presented similar performance on the maximal effort ergometer test (Fig. 2).

## Discussion

Litter size reduction soon after birth and throughout lactation has led to overweight in the neonatal period but not in youth or adulthood. Despite this, the early nutritional insult has favoured differences in haemodynamic and echocardiographic parameters later in life. The literature has previously reported related findings in adult male rats submitted to neonatal overfeeding. However, none of the studies investigated the outcomes of the same insult in female rats. According to the results here achieved, distinct outcomes may be seen in male and female rats.

As expected, the reduction of litter size leads to neonatal overweight, according to the literature, and could be addressed by the higher weight gain. Thus, this useful experimental model was validated in the present study, allowing the investigation of short- and long-term consequences of overfeeding^(26–28)^. Studies have reported that litter size reduction may increase maternal milk availability to the offspring, leading to higher body weight^(12,29–34)^. As the hypothalamic area related to food intake and satiety is not entirely structured at the beginning of the lactation period, milk intake seems to be limited only by gastrointestinal tract capacity^(35,36)^.

Litter size may modulate milk content. The literature has reported that the TAG content of the milk from dams submitted to litter reduction increases between the 10th and 21st days of lactation. Thus, neonatal overweight may also be induced by the higher energy content of maternal milk^(29,37)^.

Differences regarding food intake are also in agreement with the literature that describes hypophagia in young animals submitted to overfeeding during lactation^(38)^. Although the consequence over body mass is controversial, the similarity about feed efficiency and body weight here observed suggests the occurrence of catch-down growth, a phenomenon also reported by other studies encompassing similar animal models^(39–43)^.

The literature has correlated anthropometric markers of adiposity, systolic blood pressure and cardiovascular risk, not only in humans but also in rats^(20,44,45)^. According to the relationship ascribed, data indicate that adult male rats from reduced litters presented increased cardiovascular risk compared with regular ones. Abdominal fat deposition is related to pathological conditions and may favour atherosclerosis and acute myocardial infarction^(46)^. Although the literature has already reported the increase of blood pressure in adult male rats due to neonatal overfeeding^(26,47–50)^, the same analysis has not included female rats. Thus, data from the present study suggest that the reduction in litter size does not affect the cardiovascular risk of female animals as described for males.

Higher levels of systolic blood pressure, as seen in young and adult male rats submitted to litter size reduction, predispose to diastolic dysfunction and structural remodelling of the left ventricle, a central change in the pathogenesis of cardiac dysfunction. Indeed, echocardiographic data of the present study suggest the occurrence of myocardial hypertrophy and concentric remodelling of the left ventricle in these animals. These structural alterations may eventually lead to ventricular dilation and systolic dysfunction in heart failure progression^(51–58)^. Although changes regarding echocardiographic parameters in this animal model have not been described previously, the literature reports that overnourishment during lactation may increase cardiac sensitivity to insulin and leptin. The consequent improvement of glucose uptake and energy supply would favour cardiac hypertrophy in male rats^(59)^.

Despite no preliminary signs of cardiovascular risk increase in female rats from reduced litters, there were differences regarding echocardiographic parameters. Data suggest the occurrence of cardiac structural changes in young females. The lack of cardiac hypertrophy inferences in adulthood may be discussed, taking sexual maturation into account. Female rats reach puberty around postnatal day 30^(60)^ and reproductive senescence occurs between 15 and 20 months of age^(61)^. An oestrogen-cardioprotective effect throughout the reproductive phase is widely ascribed in many studies. This hormone can act directly on cardiac myocytes. Its negative modulatory effect on gene expression of plasma membrane Ca^2+^ channels reduces the risk of arrhythmias and other cardiovascular events. Oestrogen may also mitigate cardiac hypertrophy by increasing the expression of atrial natriuretic peptide and decreasing apoptosis/necrosis of cardiac/endothelial cells^(62–66)^.

Echocardiography data suggest that the reported structural changes are without functional impairment^(21)^. These data may explain the similar performance noticed for the animals on the maximum effort ergometer test. Exercise intolerance, the main symptom of diastolic heart failure, can be assessed by cardiopulmonary exercise tests that constitute an accurate, reliable and reproducible method that yields important outcomes^(67)^. Maximal effort ergometer tests have already been applied to assess cardiorespiratory capacity in rats^(68)^. The literature provides a linear relationship between maximum speed and O_2_ consumption^(69)^.

The present study presents a few limitations that do not allow mechanicist discussion but do not compromise data interpretation and the main findings. There was no monitoring of milk consumption, secretion and content during lactation. Thus, it is not possible to precisely explain why the reduction in litter size generated neonatal overweight. Besides, the lack of hormonal dosage makes a more detailed discussion about the cardioprotective effects of oestrogen in this experimental model somewhat speculative.

In conclusion, the present study corroborates the literature that reports an increase in cardiovascular risk in male rats due to neonatal overfeeding. It also shows that the rise of anthropometric markers of adiposity and blood pressure programme cardiac hypertrophy and concentric remodelling without functional impairment. Likewise, contributing to personalised/gender medicine, this study has shown for the first time that similar early insult in female rats promotes cardiac hypertrophy in youth without changes in biometric and haemodynamic parameters. More studies are warranted to investigate sex differences better and the underlying mechanism involved in cardiac structure preservation in adult female rats submitted to neonatal overnourishment, as well as reproductive senescence impact.

## References

[1] Mello ED, Luft VC & Meyer F (2004) Childhood obesity towards effectiveness? J Pediatr *(*Rio J*)* 80, 173–182. 15192759

[2] Balasko M, Soos S, Szekley M, (2014) Leptin and aging: review and questions with particular emphasis on its role in the central regulation of energy balance. J Chem Neuroanat 61, 248–255. 25218974 10.1016/j.jchemneu.2014.08.006

[3] Lima NDS, Caria CREP, Gambero A, (2018) The effect of guarana (*Paullinia cupana*) on metabolic and inflammatory parameters in adult male mice programmed by maternal obesity. Eur J Nutr 58, 765–774. 29626231 10.1007/s00394-018-1686-1

[4] Global Health Metrics (2017) Global, regional, and national age-sex specific mortality for 264 causes of death, 1980–2016: a systematic analysis for the Global Burden of Disease Study 2016. Lancet 390, 1151–1210. 28919116 10.1016/S0140-6736(17)32152-9 PMC5605883

[5] Barker DJ, Eriksson JG, Forsén T, (2002) Fetal origins of adult disease: strength of effects and biological basis. Int J Epidemiol 31, 1235–1239. 12540728 10.1093/ije/31.6.1235

[6] Penkler M, Hanson M, Biesma R, (2019) DOHad in science and society: emergent opportunities and novel responsibilities. J Dev Orig Health Dis 10, 268–273. 30466503 10.1017/S2040174418000892

[7] Langley-Evans SC (2015) Nutrition in early life and the programming of adult disease: a review. J Hum Nutr Diet 28, Suppl. 1, S1–S14. 10.1111/jhn.12212 24479490

[8] Pelouch V, Kolář F, Milerová M, (1997) Effect of the preweaning nutritional state on the cardiac protein profile and functional performance of the rat heart. Mol Cell Biochem 177, 221–228. 9450666 10.1023/a:1006823608341

[9] Kennedy GC (1957) The development with age of hypothalamic restraint upon the appetite of the rat. J Endocrinol 16, 9–17. 13491729 10.1677/joe.0.0160009

[10] Bei F, Jia J, Jia YQ, (2015) Long-term effect of early postnatal overnutrition on insulin resistance and serum fatty acid profiles in male rats. Lipids Health Dis 14, 96. 26302954 10.1186/s12944-015-0094-2 PMC4549095

[11] Granado M, Fernandez N, Monge L, (2013) Effects of coronary ischemia–reperfusion in a rat model of early overnutrition. Role of angiotensin receptors. PLOS ONE 8, e54984. 23383303 10.1371/journal.pone.0054984 PMC3562319

[12] Vieira AK, Soares VM, Bernardo AF, (2015) Overnourishment during lactation induces metabolic and hemodynamic heart impairment during adulthood. Nutr Metab Cardiovasc Dis 25, 1062–1069. 26315623 10.1016/j.numecd.2015.07.009

[13] Docherty JR, Stanford SC, Panattieri RA, (2019) Sex: a change in our guidelines to authors to ensure that this is no longer an ignored experimental variable. Br J Pharmacol 176, 4081–4086. 31441038 10.1111/bph.14761 PMC6877799

[14] Gerdts E & Regitz-Zagrosek V (2019) Sex differences in cardiometabolic disorders. Nat Med 25, 1657–1666. 31700185 10.1038/s41591-019-0643-8

[15] Siontis KC, Ommen SR & Geske JB (2019) Sex, survival, and cardiomyopathy: differences between men and women with hypertrophic cardiomyopathy. J Am Heart Assoc 8, e014448. 31663428 10.1161/JAHA.119.014448 PMC6898853

[16] Miller VM (2014) Why are sex and gender important to basic physiology and translational and individualized medicine? Am J Physiol Heart Circ Physiol 306, 781–788. 10.1152/ajpheart.00994.2013 PMC3949049 24414073

[17] Bailoo JD, Reichlin TS & Würbel H (2014) Refinement of experimental design and conduct in laboratory animal research. ILAR J 55, 383–391. 25541540 10.1093/ilar/ilu037

[18] Schneider M (2013) Adolescence as a vulnerable period to alter rodent behavior. Cell Tissue Res 354, 99–106. 23430475 10.1007/s00441-013-1581-2

[19] Bernadis LL & Patterson BD (1968) Correlation between ‘Lee index’ and carcass fat content in weanling and adult female rats with hypothalamic lesions. J Endocrinol 40, 527–528. 4868415 10.1677/joe.0.0400527

[20] Novelli ELB, Diniz YS, Galhardi CM, (2007) Anthropometrical parameters and markers of obesity in rats. Lab Anim 41, 111–119. 17234057 10.1258/002367707779399518

[21] Lang RM, Bierig M, Devereux RB, (2005) Chamber Quantification Writing Group; American Society of Echocardiography's Guidelines and Standards Committee; European Association of Echocardiography. Recommendations for chamber quantification: a report from the American Society of Echocardiography's Guidelines and Standards Committee and the Chamber Quantification Writing Group, developed in conjunction with the European Association of Echocardiography, a branch of the European Society of Cardiology. J Am Soc Echocardiogr 18, 1440–1463. 16376782 10.1016/j.echo.2005.10.005

[22] Johns C, Gavras I, Handy DE, (1996) Models of experimental hypertension in mice. Hypertension 28, 1064–1069. 8952597 10.1161/01.hyp.28.6.1064

[23] Fritz M & Rinaldi G (2007) Influence of nitric oxide-mediated vasodilation on the blood pressure measured with the tail-cuff method in the rat. J Biomed Sci 14, 757–765. 17634759 10.1007/s11373-007-9191-1

[24] Molnar AM, Servais S, Guichardant M, (2006) Mitochondrial H_2_O_2_ production is reduced with acute and chronic eccentric exercise in rat skeletal muscle. Antioxid Redox Signal 8, 548–558. 16677099 10.1089/ars.2006.8.548

[25] Wonders KY, Hydock DS & Hayward R (2007) Time-course of changes in cardiac function during recovery after acute exercise. Appl Physiol Nutr Metab 32, 1164–1169. 18059591 10.1139/H07-127

[26] Plagemann A, Harder T, Rake A, (1999) Perinatal elevation of hypothalamic insulin, acquired malformation of hypothalamic galaninergic neurons, and syndrome X-like alterations in adulthood of neonatally overfed rats. Brain Res 836, 146–155. 10415413 10.1016/s0006-8993(99)01662-5

[27] Sivanandam S, Sinaiko AR, Jacobs DR Jr, (2006) Relation of increase in adiposity to increase in left ventricular mass from childhood to young adulthood. Am J Cardiol 98, 411–415. 16860034 10.1016/j.amjcard.2006.02.044

[28] Habbout A, Li N, Rochette L, (2013) Postnatal overfeeding in rodents by litter size reduction induces major short- and long-term pathophysiological consequences. J Nutr 143, 553–562. 23446961 10.3945/jn.112.172825

[29] Cunha ACSR, Pereira RO, Pereira MJS, (2009) Long-term effects of overfeeding during lactation on insulin secretion the role of GLUT-2. J Nutr Biochem 20, 435–442. 18708286 10.1016/j.jnutbio.2008.05.002

[30] Faust IM, Johnson PR & Hirsch J (1980) Long-term effects of early nutritional experience on the development of obesity in the rat. J Nutr 110, 2027–2034. 7420206 10.1093/jn/110.10.2027

[31] Plagemann A, Heidrich I, Götz F, (1992) Obesity and enhanced diabetes and cardiovascular risk in adult rats due to early postnatal overfeeding. Exp Clin Endocrinol 99, 154–158. 1526266 10.1055/s-0029-1211159

[32] Heidel E, Plagemann A & Davidowa H (1999) Increased response to NPY of hypothalamic VMN neurons in postnatally overfed juvenile rats. Neuroreport 10, 1827–1831. 10501515 10.1097/00001756-199906230-00005

[33] Davidowa H & Plagemann A (2000) Decreased inhibition by leptin of hypothalamic arcuate neurons in neonatally overfed young rats. Neuroreport 11, 2795–2798. 10976965 10.1097/00001756-200008210-00037

[34] Thole AA, Rodrigues-Cunha AC, Carvalho SN, (2012) Progenitor cells and TNF-alpha involvement during morphological changes in pancreatic islets of obese mice. Tissue Cell 44, 238–248. 22537687 10.1016/j.tice.2012.04.001

[35] McMillen IC, Adam CL & Muhlhausler BS (2005) Early origins of obesity: programming the appetite regulatory system. J Physiol 565, 9–17. 15705647 10.1113/jphysiol.2004.081992 PMC1464497

[36] Rinaldi W, Ribeiro TAC, Marques AS, (2012) Effect of small litter size on the autonomic and metabolic responses of Wistar rats. Rev Nutr 25, 321–330.

[37] Assumpção RP, dos Santos FD, Andrade PMM, (2004) Effect of variation of *trans*-fatty acid in lactating rats’ diet on lipoprotein lipase activity in mammary gland, liver, and adipose tissue. Nutrition 20, 806–811. 15325692 10.1016/j.nut.2004.05.004

[38] Castro RM, Cesiana M, Oliveira L, (2014) Overnutrition in lactation: effects on somatic and sensorimotor development in rats. Neurobiologia *(*online) 77, 27–43.

[39] Nery CS, Pinheiro IL, Muniz GS, (2011) Murinometric evaluations and feed efficiency in rats from reduced litter during lactation and submitted or not to swimming exercise. Rev Bras Med Esporte 17, 49–55.

[40] Hausberger FX & Volz JE (1984) Feeding in infancy, adipose tissue cellularity and obesity. Physiol Behav 33, 81–87. 6505056 10.1016/0031-9384(84)90017-9

[41] Lambert EV & Koeslag JH (1992) No persistent effect of preweaning nutrition on postweaning food intake, feeding efficiency, or body energy stores in Long−Evans rats. Physiol Behav 52, 363–372. 1523265 10.1016/0031-9384(92)90285-a

[42] Bassett DR & Craig BW (1988) Influence of early nutrition on growth and adipose tissue characteristics in male and female rats. J Appl Physiol 64, 1249–1256. 3284871 10.1152/jappl.1988.64.3.1249

[43] Velkoska E, Cole TJ & Morris MJ (2005) Early dietary intervention: long-term effects on blood pressure, brain neuropeptide Y, and adiposity markers. Am J Physiol Endocrinol Metab 288, 1236–1243. 10.1152/ajpendo.00505.2004 15644456

[44] Radovanovic CAT, Santos LA, Carvalho MDB, (2014) Arterial hypertension and other risk factors associated with cardiovascular diseases among adults. Rev LatAm Enfermagem 22, 547–553. 10.1590/0104-1169.3345.2450 PMC4292653 25296137

[45] Abdul MK, Ur RH, Yousaf MS, (2017) Sub-chronic exposure to low concentration of dibutyl phthalate affects anthropometric parameters and markers of obesity in rats. Environ Sci Pollut Res Int 24, 25462–25467. 28823096 10.1007/s11356-017-9952-y

[46] Moura V & Monteiro R (2010) Role of adipose tissue in inflammation and metabolism of obese patients. Int J Food Sci Nutr 16, 15–22.

[47] Habbout A, Delemasure S, Goirand F, (2012) Postnatal overfeeding in rats leads to moderate overweight and to cardiometabolic and oxidative alterations in adulthood. Biochimie 94, 117–124. 21978927 10.1016/j.biochi.2011.09.023

[48] Kappeler L, De Magalhaes Filho C, Leneuve P, (2009) Early postnatal nutrition determines somatotropic function in mice. Endocrinology 150, 314–323. 18801897 10.1210/en.2008-0981

[49] Yim HE, Ha KS, Bae IS, (2012) Postnatal early overnutrition dysregulates the intrarenal renin–angiotensin system and extracellular matrix-linked molecules in juvenile male rats. J Nutr Biochem 23, 937–945. 21752621 10.1016/j.jnutbio.2011.04.020

[50] Boubred F, Daniel L, Buffat C, (2009) Early postnatal overfeeding induces early chronic renal dysfunction in adult male rats. Am J Physiol Renal Physiol 297, 943–951. 10.1152/ajprenal.90704.2008 19656908

[51] Nadruz W (2015) Myocardial remodeling in hypertension. J Hum Hypertens 29, 1–6. 24804791 10.1038/jhh.2014.36

[52] Nagueh SF, Appleton CP, Gillebert TC, (2009) Recommendations for the evaluation of left ventricular diastolic function by echocardiography. Eur J Echocardiogr 10, 165–193. 10.1093/ejechocard/jep007 19270053

[53] Grossman W & Paulus WJ (2013) Myocardial stress and hypertrophy: a complex interface between biophysics and cardiac remodeling. J Clin Invest 123, 3701–3703. 23999445 10.1172/JCI69830 PMC3754273

[54] Konstam MA, Kramer DG, Patel AR, (2011) Left ventricular remodeling in heart failure: current concepts in clinical significance and assessment. JACC Cardiovasc Imaging 4, 98–108. 21232712 10.1016/j.jcmg.2010.10.008

[55] Drazner MH (2011) The progression of hypertensive heart disease. Circulation 123, 327–334. 21263005 10.1161/CIRCULATIONAHA.108.845792

[56] Gaasch WH & Zile MR (2011) Left ventricular structural remodeling in health and disease: with special emphasis on volume, mass, and geometry. J Am Coll Cardiol 58, 1733–1740. 21996383 10.1016/j.jacc.2011.07.022

[57] Zile MR, Baicu CF & Gaasch WH (2004) Diastolic heart failure – abnormalities in active relaxation and passive stiffness of the left ventricle. N Engl J Med 350, 1953–1959. 15128895 10.1056/NEJMoa032566

[58] Zile MR, Gaasch WH, Carroll JD, (2001) Heart failure with a normal ejection fraction: is measurement of diastolic function necessary to make the diagnosis of diastolic heart failure? Circulation 104, 779–782. 11502702 10.1161/hc3201.094226

[59] Pereira RO, Moreira AS, de Carvalho L, (2006) Overfeeding during lactation modulates insulin and leptin signaling cascade in rats’ hearts. Regul Pept 136, 117–121. 16806530 10.1016/j.regpep.2006.05.002

[60] Leibowitz SF, Akabayashi A, Alexander J, (2009) Puberty onset in female rats: relationship with fat intake, ovarian steroids and the peptides, galanin and enkephalin, in the paraventricular and medial preoptic nuclei. J Neuroendocrinol 21, 538–549. 19500224 10.1111/j.1365-2826.2009.01870.x PMC2782789

[61] Sengupta P (2013) The laboratory rat: relating its age with human's. Int J Prev Med 4, 624–630. 23930179 PMC3733029

[62] Kuller LH, Meilahn EN, Cauley JA, (1994) Epidemiologic studies of menopause: changes in risk factors and disease. Exp Gerontol 29, 495–509. 7925767 10.1016/0531-5565(94)90030-2

[63] Machi JF, Dias DS, Freitas SC, (2016) Impact of aging on cardiac function in a female rat model of menopause: role of autonomic control, inflammation, and oxidative stress. Clin Interv Aging 11, 341–350. 27042032 10.2147/CIA.S88441 PMC4809309

[64] Braz GRF, Emiliano AS, Sousa SM, (2017) Maternal low-protein diet in female rat heart: possible protective effect of estradiol. J Dev Orig Health Dis 8, 322–330. 28264755 10.1017/S2040174417000058

[65] Silva MCR, Arandas MJG, Lima-Junior NB, (2016) Histomorphometric analysis of cardiomyocytes and collagen deposition in the heart muscle of ovariectomized rats. Pesq Vet Bras 36, 216–220.

[66] Liu A, Gao L, Kang S, (2012) Testosterone enhances estradiol's cardioprotection in ovariectomized rats. J Endocrinol 212, 61–69. 21965546 10.1530/JOE-11-0181

[67] Kitzman DW & Groban L (2008) Exercise intolerance. Heart Fail Clin 4, 99–115. 18313628 10.1016/j.hfc.2007.12.002 PMC2700357

[68] Marques EB, Rocha NN, dos Santos MC, (2015) Cardiac programming in rats submitted to leptin treatment during lactation. Int J Cardiol 181, 141–143. 25497539 10.1016/j.ijcard.2014.12.015

[69] Rodrigues B, Figueroa DM, Mostarda CT, (2007) Maximal exercise test is a useful method for physical capacity and oxygen consumption determination in streptozotocin-diabetic rats. Cardiovasc Diabetol 13, 6–38. 10.1186/1475-2840-6-38 PMC2222609 18078520

